# Bis(2-amino-4-methyl­pyridinium) bis­(pyridine-2,6-dicarboxyl­ato)cuprate(II)

**DOI:** 10.1107/S1600536811001139

**Published:** 2011-01-15

**Authors:** Hossein Aghabozorg, Azadeh Mofidi Rouchi, Behrouz Notash, Masoud Mirzaei

**Affiliations:** a Faculty of Chemistry, Islamic Azad University, North Tehran Branch, Tehran, Iran; b Department of Chemistry, Shahid Beheshti University, G. C., Evin, Tehran, 1983963113, Iran; c Department of Chemistry, School of Sciences, Ferdowsi University of Mashhad, Mashhad 917791436, Iran

## Abstract

The asymmetric unit of the title compound, (C_6_H_9_N_2_)_2_[Cu(C_7_H_3_NO_4_)_2_], contains half of a [Cu(pydc)_2_]^2−^ (pydcH_2_ is pyridine-2,6-dicarb­oxy­lic acid) anion and one protonated 2-amino-4-methyl­pyridine (2a4mpH)^+^ counter-ion. The anion is a six-coordinated complex with a distorted CuN_2_O_4_ octa­hedral geometry around the Cu^II^ ion. N—H⋯O and C—H⋯O hydrogen bonds along with π–π contacts between the pyridine rings of the (2a4mpH)^+^ cations [centroid–centroid distance = 3.573 (2) Å] stabilize the crystal structure.

## Related literature

For background to proton-transfer compounds, see: Aghabozorg *et al.* (2008 ▶). For related structures see: Aghabozorg *et al.* (2011 ▶); Eshtiagh-Hosseini, Aghabozorg *et al.* (2010 ▶); Eshtiagh-Hosseini, Gschwind *et al.* (2010 ▶); Sharif *et al.* (2010 ▶).
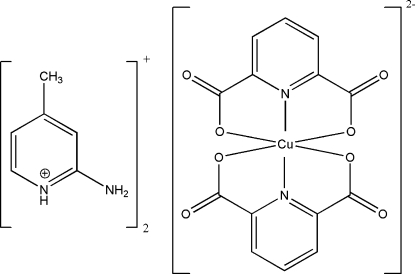


## Experimental

### 

#### Crystal data

(C_6_H_9_N_2_)_2_[Cu(C_7_H_3_NO_4_)_2_]
*M* *_r_* = 612.06
Monoclinic, 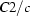

*a* = 24.034 (5) Å
*b* = 14.231 (3) Å
*c* = 7.9780 (16) Å
β = 107.01 (3)°
*V* = 2609.3 (10) Å^3^
*Z* = 4
Mo *K*α radiation
μ = 0.9 mm^−1^
*T* = 298 K
0.45 × 0.15 × 0.10 mm

#### Data collection

Stoe IPDS II diffractometer
Absorption correction: numerical [shape of crystal determined optically (*X-RED32*; Stoe & Cie, 2005 ▶)] *T*_min_ = 0.743, *T*_max_ = 0.846
8829 measured reflections
3509 independent reflections
2785 reflections with *I* > 2σ(*I*)
*R*_int_ = 0.061

#### Refinement

*R*[*F* ^2^ > 2σ(*F* ^2^)] = 0.057
*wR*(*F* ^2^) = 0.119
*S* = 1.15
3509 reflections
201 parameters
H atoms treated by a mixture of independent and constrained refinement
Δρ_max_ = 0.39 e Å^−3^
Δρ_min_ = −0.30 e Å^−3^

### 

Data collection: *X-AREA* (Stoe & Cie, 2005 ▶); cell refinement: *X-AREA*; data reduction: *X-AREA*; program(s) used to solve structure: *SHELXS97* (Sheldrick, 2008 ▶); program(s) used to refine structure: *SHELXL97* (Sheldrick, 2008 ▶); molecular graphics: *ORTEP-3 for Windows* (Farrugia, 1997 ▶); software used to prepare material for publication: *WinGX* (Farrugia, 1999 ▶).

## Supplementary Material

Crystal structure: contains datablocks I, global. DOI: 10.1107/S1600536811001139/bt5449sup1.cif

Structure factors: contains datablocks I. DOI: 10.1107/S1600536811001139/bt5449Isup2.hkl

Additional supplementary materials:  crystallographic information; 3D view; checkCIF report

## Figures and Tables

**Table 1:** Hydrogen-bond geometry (Å, °)

*D*—H⋯*A*	*D*—H	H⋯*A*	*D*⋯*A*	*D*—H⋯*A*
N3—H3*A*⋯O2^i^	0.92 (4)	1.77 (4)	2.662 (4)	163 (4)
N4—H4*A*⋯O1^i^	0.85 (5)	2.22 (5)	3.056 (4)	170 (4)
N4—H4*B*⋯O3	0.89 (5)	1.97 (5)	2.854 (4)	176 (4)
C7—H7⋯O1^ii^	0.93	2.58	3.250 (4)	130
C14—H14⋯O4^iii^	0.93	2.42	3.160 (4)	136

Symmetry codes: (i) 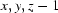; (ii) 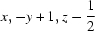; (iii) 
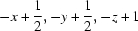
.

**Fig. 1.**
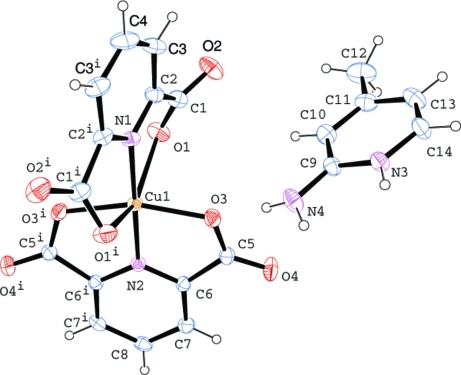
The molecular structure of (2a4mpH)+2.[Cu(pydc)2] with displacement ellipsoids drawn at 30% probability level (symmetry code: i: -x, y, 3/2 - z).

**Fig. 2.**
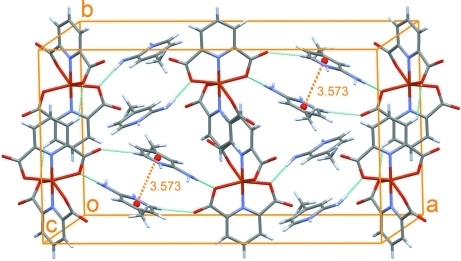
The packing diagram of (2a4mpH)+2.[Cu(pydc)2]. The intermolecular N—H···O and C—H···O hydrogen bonds and π-π contacts are shown as blue and orange dashed lines, respectively.

## supplementary crystallographic information

### Comment

Polycarboxylate ligands are widely applied to assemble supramolecular network decorated by coordination bonds, van der Waals interactions, and π –π stacking. Due to the manifold N– and O-donors of pyridine or pyrazine-(di)carboxylic ligands, metal pyridine- or pyrazine dicarboxylates can contrast versatile structural motifs, which finally aggregate to generate various supramolecular architectures with interesting properties. As ones of the dicarboxylate ligands, pydcH_2_ have drawn extensive attentions. Continuing with our previous works on synthesizing coordination and proton transfer compounds (Aghabozorg *et al.* 2008, 2011), (Eshtiagh-Hosseini, Aghabozorg *et al.*, 2010, Eshtiagh-Hosseini, Gschwind *et al.*, 2010), (Sharif *et al.*, 2010), herein, we planned the reaction between pydcH_2_, 2a4mp, and copper^II^ nitrate trihydrate which resulted in the formation of (2a4mpH)^+^_2_.[Cu(pydc)_2_] crystals (Fig. 1). Crystal packing diagram related to the title compound is also rendered in the Fig. 2. In the anionic fragment, the Cu^II^ atom is six-coordinated by two nitrogen and four oxygen atoms from the carboxylate groups of two (pydc)^2-^ ligands, with bond length ranges of 1.911 (3)–2.029 (2) Å. The N1—Cu1—N2 [180.000 (1)°], O1—Cu1—O1 [146.67 (5)°] and O3—Cu1—O3 [160.23 (5)°] angles. The coordination environment around Cu^II^ is distorted octahedral. In the crystal structure of the title compound, there are intermolecular C—H···O and N—H···O hydrogen bonds (Table 1) and also π-π contacts between pyridine rings of (2a4mpH)^+^ with centroid-centroid distance *Cg*1···*Cg*1^i^ equel to 3.573 (2) Å [symmetry code: (i) 2 - *x*, 2 - *y*,1 - *z*, where *Cg*1 is the centroid of ring N3/C9—C11/C13—C14]. (Fig. 2) stabilize the structure.

### Experimental

A solution of pyridine-2,6-dicarboxylic acid (pydcH_2_) (167 mg, 1 mmol) in 10 ml me thanol was added to a solution of 2-amino-4-methylpyridine (2a4mp) (216 mg, 0.6 mmol) in 10 ml me thanol and stirred for 4 hrs. Then a solution of Cu(NO_3_)_2_.3H_2_O (240 mg, 1 mmol) in 3 ml me thanol was added to the solution of pydcH_2_ and 2a4mp. To the resulted precipitate was added 1 ml of DMSO and stirred for several minutes under heating. By slove evaporation of this solution in room temprature, green crystals of the title compound were obtained after three week which were suitable for X-ray analysis (m.p 265–267 °C).

### Refinement

The hydrogen atoms of the N—H and NH_2_ groups were found in a difference Fourier map and refined isotropically without restraint. The C—H protons were positioned geometrically and refined as riding atoms with C—H = 0.93 Å and *U*iso(H) = 1.2 *U*eq(C) for aromatic C—H groups and C—H = 0.96 Å and *U*iso(H) = 1.5 *Ueq*(C) for methyl group.

### Crystal data

**Table:**

(C_6_H_9_N_2_)_2_[Cu(C_7_H_3_NO_4_)_2_]	*F*(000) = 1260
*M**_r_* = 612.06	*D*_x_ = 1.558 Mg m^−^^3^
Monoclinic, *C*2/*c*	Mo *K*α radiation, λ = 0.71073 Å
Hall symbol: -C 2yc	Cell parameters from 3509 reflections
*a* = 24.034 (5) Å	θ = 2.9–29.2°
*b* = 14.231 (3) Å	µ = 0.9 mm^−^^1^
*c* = 7.9780 (16) Å	*T* = 298 K
β = 107.01 (3)°	Needle, blue
*V* = 2609.3 (10) Å^3^	0.45 × 0.15 × 0.1 mm
*Z* = 4	

### Data collection

**Table:**

Stoe IPDS II diffractometer	3509 independent reflections
Radiation source: fine-focus sealed tube	2785 reflections with *I* > 2σ(*I*)
graphite	*R*_int_ = 0.061
Detector resolution: 0.15 mm pixels mm^-1^	θ_max_ = 29.2°, θ_min_ = 2.9°
rotation method scans	*h* = −30→32
Absorption correction: numerical [shape of crystal determined optically (*X-RED32*, Stoe & Cie, 2005)]	*k* = −19→16
*T*_min_ = 0.743, *T*_max_ = 0.846	*l* = −10→10
8829 measured reflections	

### Refinement

**Table:**

Refinement on *F*^2^	Primary atom site location: structure-invariant direct methods
Least-squares matrix: full	Secondary atom site location: difference Fourier map
*R*[*F*^2^ > 2σ(*F*^2^)] = 0.057	Hydrogen site location: inferred from neighbouring sites
*wR*(*F*^2^) = 0.119	H atoms treated by a mixture of independent and constrained refinement
*S* = 1.15	*w* = 1/[σ^2^(*F*_o_^2^) + (0.0336*P*)^2^ + 5.0605*P*] where *P* = (*F*_o_^2^ + 2*F*_c_^2^)/3
3509 reflections	(Δ/σ)_max_ < 0.001
201 parameters	Δρ_max_ = 0.39 e Å^−^^3^
0 restraints	Δρ_min_ = −0.30 e Å^−^^3^

### Special details

**Table:**

Geometry. All e.s.d.'s (except the e.s.d. in the dihedral angle between two l.s. planes) are estimated using the full covariance matrix. The cell e.s.d.'s are taken into account individually in the estimation of e.s.d.'s in distances, angles and torsion angles; correlations between e.s.d.'s in cell parameters are only used when they are defined by crystal symmetry. An approximate (isotropic) treatment of cell e.s.d.'s is used for estimating e.s.d.'s involving l.s. planes.
Refinement. Refinement of *F*^2^ against ALL reflections. The weighted *R*-factor *wR* and goodness of fit *S* are based on *F*^2^, conventional *R*-factors *R* are based on *F*, with *F* set to zero for negative *F*^2^. The threshold expression of *F*^2^ > σ(*F*^2^) is used only for calculating *R*-factors(gt) *etc*. and is not relevant to the choice of reflections for refinement. *R*-factors based on *F*^2^ are statistically about twice as large as those based on *F*, and *R*- factors based on ALL data will be even larger.

### Fractional atomic coordinates and isotropic or equivalent isotropic displacement parameters (Å^2^)

**Table:**

	*x*	*y*	*z*	*U*_iso_*/*U*_eq_	
C2	0.04362 (14)	0.1040 (2)	0.8614 (4)	0.0350 (6)	
O2	0.13708 (12)	0.1246 (2)	1.0589 (3)	0.0592 (7)	
O1	0.07172 (11)	0.23932 (17)	1.0341 (3)	0.0475 (6)	
C7	0.04400 (13)	0.5659 (2)	0.7068 (4)	0.0338 (6)	
H7	0.0734	0.5980	0.6770	0.041*	
C8	0.0000	0.6143 (3)	0.7500	0.0359 (9)	
H8	0.0000	0.6796	0.7500	0.043*	
Cu1	0.0000	0.29007 (3)	0.7500	0.02835 (14)	
N2	0.0000	0.4244 (2)	0.7500	0.0266 (6)	
N4	0.11686 (12)	0.2123 (2)	0.4309 (4)	0.0461 (7)	
O3	0.07121 (9)	0.31454 (14)	0.6690 (3)	0.0370 (5)	
N3	0.19892 (12)	0.14346 (19)	0.3935 (4)	0.0388 (6)	
N1	0.0000	0.1521 (2)	0.7500	0.0318 (7)	
C9	0.17056 (13)	0.1785 (2)	0.5020 (4)	0.0345 (6)	
C11	0.25320 (16)	0.1393 (2)	0.7462 (4)	0.0444 (7)	
C10	0.19805 (15)	0.1743 (2)	0.6831 (4)	0.0420 (7)	
H10	0.1786	0.1957	0.7608	0.050*	
O4	0.12832 (10)	0.43226 (18)	0.6314 (3)	0.0493 (6)	
C6	0.04298 (11)	0.46879 (19)	0.7094 (3)	0.0272 (5)	
C5	0.08574 (12)	0.4019 (2)	0.6666 (4)	0.0320 (6)	
C13	0.28131 (16)	0.1052 (3)	0.6265 (5)	0.0516 (9)	
H13	0.3188	0.0808	0.6665	0.062*	
C14	0.25339 (15)	0.1082 (3)	0.4529 (5)	0.0485 (8)	
H14	0.2719	0.0859	0.3734	0.058*	
C12	0.2851 (2)	0.1384 (3)	0.9394 (5)	0.0679 (12)	
H12A	0.3097	0.1927	0.9684	0.102*	
H12B	0.3084	0.0826	0.9675	0.102*	
H12C	0.2574	0.1393	1.0052	0.102*	
C1	0.08860 (15)	0.1616 (2)	0.9957 (4)	0.0395 (7)	
C3	0.04539 (18)	0.0064 (2)	0.8605 (5)	0.0485 (8)	
H3	0.0770	−0.0255	0.9339	0.058*	
C4	0.0000	−0.0425 (3)	0.7500	0.0591 (15)	
H4	0.0000	−0.1079	0.7500	0.071*	
H3A	0.1810 (18)	0.148 (3)	0.275 (5)	0.057 (11)*	
H4B	0.1012 (19)	0.245 (3)	0.501 (6)	0.062 (12)*	
H4A	0.108 (2)	0.225 (3)	0.322 (7)	0.074 (15)*	

### Atomic displacement parameters (Å^2^)

**Table:**

	*U*^11^	*U*^22^	*U*^33^	*U*^12^	*U*^13^	*U*^23^
C2	0.0432 (17)	0.0305 (14)	0.0316 (14)	0.0044 (12)	0.0116 (13)	−0.0005 (11)
O2	0.0498 (15)	0.0694 (18)	0.0477 (14)	0.0226 (13)	−0.0024 (12)	−0.0063 (12)
O1	0.0519 (14)	0.0415 (13)	0.0427 (12)	0.0065 (11)	0.0036 (11)	−0.0087 (10)
C7	0.0291 (14)	0.0305 (14)	0.0372 (14)	−0.0073 (11)	0.0026 (12)	0.0014 (11)
C8	0.033 (2)	0.0240 (18)	0.042 (2)	0.000	−0.0011 (18)	0.000
Cu1	0.0325 (3)	0.0218 (2)	0.0336 (2)	0.000	0.01408 (19)	0.000
N2	0.0261 (16)	0.0261 (15)	0.0273 (15)	0.000	0.0072 (13)	0.000
N4	0.0400 (14)	0.0526 (17)	0.0444 (15)	0.0111 (14)	0.0100 (12)	−0.0122 (15)
O3	0.0376 (11)	0.0338 (11)	0.0444 (11)	0.0037 (9)	0.0193 (10)	−0.0036 (9)
N3	0.0367 (14)	0.0414 (14)	0.0378 (13)	0.0074 (11)	0.0102 (11)	−0.0009 (11)
N1	0.042 (2)	0.0233 (15)	0.0304 (16)	0.000	0.0112 (15)	0.000
C9	0.0320 (14)	0.0303 (13)	0.0410 (15)	−0.0003 (11)	0.0104 (12)	−0.0066 (11)
C11	0.0478 (18)	0.0351 (16)	0.0440 (17)	−0.0024 (14)	0.0033 (14)	−0.0012 (14)
C10	0.0445 (18)	0.0415 (16)	0.0399 (16)	0.0009 (14)	0.0120 (14)	−0.0061 (13)
O4	0.0357 (12)	0.0546 (14)	0.0658 (15)	−0.0093 (11)	0.0279 (12)	−0.0084 (12)
C6	0.0249 (13)	0.0304 (13)	0.0254 (11)	−0.0025 (10)	0.0059 (10)	−0.0004 (10)
C5	0.0304 (14)	0.0357 (14)	0.0305 (13)	−0.0020 (11)	0.0098 (11)	−0.0030 (11)
C13	0.0375 (18)	0.049 (2)	0.062 (2)	0.0101 (15)	0.0032 (16)	−0.0013 (16)
C14	0.0394 (18)	0.051 (2)	0.058 (2)	0.0144 (15)	0.0181 (16)	−0.0010 (16)
C12	0.080 (3)	0.058 (2)	0.049 (2)	0.000 (2)	−0.008 (2)	0.0008 (18)
C1	0.0470 (18)	0.0404 (17)	0.0294 (14)	0.0049 (14)	0.0086 (13)	−0.0005 (12)
C3	0.065 (2)	0.0306 (15)	0.0484 (18)	0.0147 (15)	0.0148 (17)	0.0050 (13)
C4	0.088 (4)	0.022 (2)	0.067 (3)	0.000	0.022 (3)	0.000

### Geometric parameters (Å, °)

**Table:**

C2—N1	1.346 (3)	N3—C14	1.351 (4)
C2—C3	1.390 (4)	N3—H3A	0.92 (4)
C2—C1	1.520 (4)	N1—C2^i^	1.346 (3)
O2—C1	1.243 (4)	C9—C10	1.403 (4)
O1—C1	1.246 (4)	C11—C10	1.367 (5)
C7—C6	1.382 (4)	C11—C13	1.407 (5)
C7—C8	1.387 (4)	C11—C12	1.507 (5)
C7—H7	0.9300	C10—H10	0.9300
C8—C7^i^	1.387 (4)	O4—C5	1.217 (3)
C8—H8	0.9300	C6—C5	1.511 (4)
Cu1—N2	1.911 (3)	C13—C14	1.352 (5)
Cu1—N1	1.964 (3)	C13—H13	0.9300
Cu1—O3	2.029 (2)	C14—H14	0.9300
Cu1—O3^i^	2.029 (2)	C12—H12A	0.9600
N2—C6	1.330 (3)	C12—H12B	0.9600
N2—C6^i^	1.330 (3)	C12—H12C	0.9600
N4—C9	1.338 (4)	C3—C4	1.374 (5)
N4—H4B	0.89 (5)	C3—H3	0.9300
N4—H4A	0.85 (5)	C4—C3^i^	1.374 (5)
O3—C5	1.292 (4)	C4—H4	0.9300
N3—C9	1.344 (4)		
			
N1—C2—C3	121.6 (3)	C10—C11—C12	121.8 (3)
N1—C2—C1	116.5 (3)	C13—C11—C12	119.3 (3)
C3—C2—C1	121.8 (3)	C11—C10—C9	120.5 (3)
C6—C7—C8	118.3 (3)	C11—C10—H10	119.7
C6—C7—H7	120.8	C9—C10—H10	119.7
C8—C7—H7	120.8	N2—C6—C7	119.8 (3)
C7^i^—C8—C7	120.5 (4)	N2—C6—C5	112.5 (2)
C7^i^—C8—H8	119.8	C7—C6—C5	127.6 (3)
C7—C8—H8	119.8	O4—C5—O3	126.4 (3)
N2—Cu1—N1	180.000 (1)	O4—C5—C6	120.1 (3)
N2—Cu1—O3	80.12 (6)	O3—C5—C6	113.5 (2)
N1—Cu1—O3	99.88 (6)	C14—C13—C11	119.4 (3)
N2—Cu1—O3^i^	80.12 (6)	C14—C13—H13	120.3
N1—Cu1—O3^i^	99.88 (6)	C11—C13—H13	120.3
O3—Cu1—O3^i^	160.24 (12)	N3—C14—C13	120.7 (3)
C6—N2—C6^i^	123.2 (3)	N3—C14—H14	119.6
C6—N2—Cu1	118.39 (17)	C13—C14—H14	119.6
C6^i^—N2—Cu1	118.39 (17)	C11—C12—H12A	109.5
C9—N4—H4B	117 (3)	C11—C12—H12B	109.5
C9—N4—H4A	116 (3)	H12A—C12—H12B	109.5
H4B—N4—H4A	120 (4)	C11—C12—H12C	109.5
C5—O3—Cu1	115.23 (17)	H12A—C12—H12C	109.5
C9—N3—C14	122.2 (3)	H12B—C12—H12C	109.5
C9—N3—H3A	118 (3)	O2—C1—O1	127.6 (3)
C14—N3—H3A	120 (3)	O2—C1—C2	116.4 (3)
C2^i^—N1—C2	118.9 (4)	O1—C1—C2	115.9 (3)
C2^i^—N1—Cu1	120.56 (18)	C4—C3—C2	119.3 (3)
C2—N1—Cu1	120.56 (18)	C4—C3—H3	120.3
N4—C9—N3	118.0 (3)	C2—C3—H3	120.3
N4—C9—C10	123.8 (3)	C3^i^—C4—C3	119.2 (4)
N3—C9—C10	118.2 (3)	C3^i^—C4—H4	120.4
C10—C11—C13	118.8 (3)	C3—C4—H4	120.4
			
C6—C7—C8—C7^i^	−0.48 (18)	Cu1—N2—C6—C7	179.50 (19)
O3—Cu1—N2—C6	−2.65 (14)	C6^i^—N2—C6—C5	−179.4 (2)
O3^i^—Cu1—N2—C6	177.35 (14)	Cu1—N2—C6—C5	0.6 (2)
O3—Cu1—N2—C6^i^	177.35 (14)	C8—C7—C6—N2	1.0 (4)
O3^i^—Cu1—N2—C6^i^	−2.65 (14)	C8—C7—C6—C5	179.7 (2)
N2—Cu1—O3—C5	4.56 (19)	Cu1—O3—C5—O4	176.0 (3)
N1—Cu1—O3—C5	−175.44 (19)	Cu1—O3—C5—C6	−5.4 (3)
O3^i^—Cu1—O3—C5	4.56 (19)	N2—C6—C5—O4	−178.1 (2)
C3—C2—N1—C2^i^	1.8 (2)	C7—C6—C5—O4	3.2 (5)
C1—C2—N1—C2^i^	−174.4 (3)	N2—C6—C5—O3	3.2 (3)
C3—C2—N1—Cu1	−178.2 (2)	C7—C6—C5—O3	−175.5 (3)
C1—C2—N1—Cu1	5.6 (3)	C10—C11—C13—C14	0.3 (5)
O3—Cu1—N1—C2^i^	−114.82 (16)	C12—C11—C13—C14	−178.2 (4)
O3^i^—Cu1—N1—C2^i^	65.18 (16)	C9—N3—C14—C13	1.3 (5)
O3—Cu1—N1—C2	65.18 (16)	C11—C13—C14—N3	−0.1 (6)
O3^i^—Cu1—N1—C2	−114.82 (16)	N1—C2—C1—O2	−157.6 (3)
C14—N3—C9—N4	179.6 (3)	C3—C2—C1—O2	26.2 (5)
C14—N3—C9—C10	−2.6 (5)	N1—C2—C1—O1	24.9 (4)
C13—C11—C10—C9	−1.6 (5)	C3—C2—C1—O1	−151.3 (3)
C12—C11—C10—C9	176.8 (3)	N1—C2—C3—C4	−3.6 (5)
N4—C9—C10—C11	−179.6 (3)	C1—C2—C3—C4	172.4 (3)
N3—C9—C10—C11	2.7 (5)	C2—C3—C4—C3^i^	1.7 (2)
C6^i^—N2—C6—C7	−0.50 (19)		

Symmetry codes: (i) −*x*, *y*, −*z*+3/2.

### Hydrogen-bond geometry (Å, °)

**Table:**

*D*—H···*A*	*D*—H	H···*A*	*D*···*A*	*D*—H···*A*
N3—H3A···O2^ii^	0.92 (4)	1.77 (4)	2.662 (4)	163 (4)
N4—H4A···O1^ii^	0.85 (5)	2.22 (5)	3.056 (4)	170 (4)
N4—H4B···O3	0.89 (5)	1.97 (5)	2.854 (4)	176 (4)
C7—H7···O1^iii^	0.93	2.58	3.250 (4)	130
C14—H14···O4^iv^	0.93	2.42	3.160 (4)	136

Symmetry codes: (ii) *x*, *y*, *z*−1; (iii) *x*, −*y*+1, *z*−1/2; (iv) −*x*+1/2, −*y*+1/2, −*z*+1.
